# Influence of Life Meaning on Subjective Well-Being of Older People: Serial Multiple Mediation of Exercise Identification and Amount of Exercise

**DOI:** 10.3389/fpubh.2021.515484

**Published:** 2021-07-08

**Authors:** Qi Zhang, Yang Yang, Guo-Li Zhang

**Affiliations:** School of Psychology, Beijing Sport University, Beijing, China

**Keywords:** subjective well-being, amount of exercise, exercise identification, life meaning, older people

## Abstract

**Objective:** This study aimed to explore the relationship between life meaning and subjective well-being among older people and the mediating role of subjective exercise identification and objective amount of exercise.

**Methods:** A total of 352 older respondents completed four questionnaires: the Chinese life meaning scale, the University of Newfoundland Happiness Scale, the physical activity habits questionnaire, and the exercise identification questionnaire designed for this study.

**Results:** Gender differences existed in the respondents' perception of life meaning, and men had a better perception of life meaning (*t* = 2.28, *SE* = 0.63, *p* < 0.05). There were age differences in the subjective well-being of older people (*F* = 5.69, *partial* η^2^ = 0.03, *p* < 0.01); the subjective well-being of older individuals steadily declines with rising age. Life meaning not only directly affected the subjective well-being of the respondents but also indirectly influenced their subjective well-being through the following three pathways: life meaning → exercise identification → subjective well-being (mediating effect of 22%); life meaning → amount of exercise → subjective well-being (mediating effect of 22%); and life meaning → exercise identification → amount of exercise → subjective well-being (serial multiple mediation effect of 27%).

**Conclusion:** The more positive the life meaning perception of older people is, the higher their experience of subjective well-being. For older people to sense the meaning of life, we recommend that they realize the importance of physical activities and take the initiative to engage in physical activities to obtain higher subjective well-being.

## Introduction

According to recent data from the National Bureau of Statistics of China (1), the number of people in China has exceeded 240 million, accounting for 17.3% of the total population, and the number of people aged 65 years or above is nearly 160 million, representing 11.4% of the total population. It is generally believed that a society is aging when people aged over 60 years account for 10% of the total population in a given country (2), implying that China has become an aging society. Thus, it is important to pay attention to the quality of life and subjective well-being of older people in China.

Subjective well-being refers to individuals' overall evaluation of their quality of life at a certain stage based on the selected cognitive and emotional standards they set (3). Specifically, the cognitive component refers to life satisfaction, and the emotional component includes both positive and negative emotions. Life meaning is also known as individual meaning. Steger et al. (4) define it as individuals' perceptions of themselves as human beings, the essence of their existence, and the things that are the most important to them. It includes two dimensions: existential meaning and search for meaning. Existential meaning is the degree to which an individual perceives his or her life as meaningful, while the search for meaning is the degree to which an individual actively searches for the meaning of life and emphasizes the process (5). Both life meaning and subjective well-being are regarded as the most important components of a happy life (6), important qualities of an individual's positive psychology and the cornerstone of a better life (7). Moreover, life meaning can affect subjective well-being (8). Specifically, individuals with a sense of life meaning exhibit enhanced positive emotions (9), such as happiness (10). Life meaning is closely related to subjective well-being, and they may compensate for each other in adverse circumstances (11). Furthermore, older people with a high life significance score hold optimistic and positive attitudes toward life and are less likely to feel lonely (12).

Individuals who pursue the meaning of life are aware of the value of exercise and take the initiative, especially physical exercise (13). Physical exercise refers to purposeful, planned, and regular physical activities aimed at being fit and strengthening the mind (14). The meaning of life is composed of purpose, meaning, and consistency (15), corresponding to the definition of physical exercise. Physical exercise refers to purposeful, planned, and regular physical activities. Therefore, there may be a positive association between life meaning and engagement in regular physical exercise. Individual attitudes and identification with physical exercise are highly correlated with physical exercise behavior (16), and individual identification with physical exercise is associated with subjective well-being (17). In addition, Li et al. (18) identified a significant correlation between physical exercise behavior and the happiness index in older people. Therefore, an individual's physical exercise routine is expected to be facilitated by an enhanced understanding of life. Engagement in sports can increase older people's happiness and positive feelings and reduce depression and anxiety (19). Thus, the influence of life meaning on subjective well-being may be mediated by the amount of exercise an individual performs and exercise identification.

China, as the largest population on earth, has a rapidly aging population (20). Life meaning has a positive association with subjective well-being. However, few studies have explained how life meaning affects subjective well-being from the exercise psychology perspective. As physical exercise can benefit older people physically and psychologically, two exercise-related factors were considered to explain the relation between life meaning and subjective well-being. Thus, the present study explored the direct and indirect effects of life meaning, exercise identification, and amount of exercise on subjective well-being. The present study hypothesized that life meaning can positively predict subjective well-being. Moreover, according to the literature reviewed above, a serial multiple mediation model of exercise identification and amount of exercise in the association between life meaning and subjective well-being among older people is hypothesized.

## Methods

### Participants and Procedures

The economy of Yueyang, Hunan Province, is at the middle level in China and is representative of most cities. The participants were recruited through advertisements posted in locations in Yueyang frequented by older people, including exercise areas (e.g., parks and bulletin boards) and informal spaces (e.g., chess and card rooms, activity centers, and universities for older people). 1 week after the advertisements were released, the principal investigator and the research assistant interviewed older people who volunteered to participate. The questionnaire was self-administered. For older people who could not complete the questionnaire independently due to eyesight problems, the data were collected by interview. The principal investigator or research assistant read the questionnaire verbatim to the participants with poor vision and entered the participants' answers into the questionnaire. The participants who were not visually impaired filled out the questionnaire in person and returned it. Data from the self-administered and investigator-administered questionnaires were statistically compared, and no difference between the two groups was detected. Participants with cognitive impairment or speech problems were excluded. Non-Chinese individuals were excluded. And invalid responses were removed.

The present study received ethics approval from the Kinetic Science Experiment Ethics Committee of Beijing Sport University. Participation was totally voluntary, and responses were anonymous. All the information collected was stored securely. A participant information sheet was provided at the start of the data collection. Completion of the questionnaire indicated consent.

### Measures

Chinese life meaning scale. The meaning of life questionnaire created by Steger et al. (4) was translated into Chinese and adapted by Wang and Dai et al. It measures two factors: life meaning experience and life meaning pursuit. When administered to Chinese college students, the questionnaire showed excellent internal consistency; the alpha coefficients of the two factors of life meaning experience and life meaning pursuit were 0.85 and 0.82, respectively, and the retest reliability 1 week later was 0.71.

University of Newfoundland Happiness Scale. Liu and Gong (21) revised the University of the Newfoundland Happiness Scale developed by Kozma and Stones and applied it to older people. The internal consistency coefficient of the whole scale was 0.87, and the internal consistency coefficient of each subscale was >0.80 with good reliability; thus, this scale can be used to assess the subjective well-being of older people in China. Among the 24 items in the scale, five items reflect positive emotions (PA) and five items reflect negative emotions (NA). Seven items reflect positive experiences (PE), and seven items reflect negative experiences (NE). The total score was obtained using the following formula: total happiness = PA-NA-NE+24, and the possible score ranges from 0 to 48.

Physical Activity Habits Questionnaire. This questionnaire was revised by Song based on the Physical Exercise Rating Questionnaire revised by Liang (22); the retest reliability of the scale was 0.82. The questionnaire assesses exercise habits in terms of participation in physical exercise programs, exercise intensity, exercise frequency, duration of participation in regular exercise, organizational form of participation in physical exercise, and whether participation in physical exercise is voluntary. The amount of exercise is calculated in three dimensions, namely, exercise time, exercise intensity, and exercise frequency, and the score is calculated as follows: amount of exercise = physical exercise intensity × (time-1) × frequency. Each aspect is divided into five levels, with a score of 1–5, and the possible score ranges from 0 to 100.

Exercise Identification Questionnaire. Three questions were designed in this survey to assess older people's attitudes toward exercise, their willingness to participate in physical exercise, and their judgment of the benefits of physical exercise, all scoring four points. The results revealed a moderate correlation among the three questions (*r* > 0.48, *p* < 0.01). Principal component analysis showed that the three questions could be combined into one factor with a total explanatory power of up to 71.5% and an internal consistency coefficient of 0.79; this factor was eventually named “exercise identification.”

### Statistical Analysis

The SPSS 18.0 software and Amos 21.0 software were used to analyze the data. The data were inspected for common method bias using the Harman's single-factor test. Analysis was conducted focusing on differences in lifemeaning and subjective well-being among older people in different groups. The correlation between continuous variables was examined. Hierarchical regression analysis was then conducted to determine the factors influencing life meaning and subjective well-being. The mediating effects of exercise identification and amount of exercise were evaluated using Amos 21.0, including serial multiple mediation pathway analysis.

## Results

Three hundred and fifty-two completed questionnaires were obtained. Of the 352 respondents, 143 were men (40.63%). Regarding age, 134 people were aged 61–65 years (38.00%), 109 were aged 66–70 years (31.00%), and 109 were aged over 70 years (31.00%).

### Common Method Deviation Control and Inspection

Data collection by survey questionnaires may produce a common method bias effect. Thus, a Harman single-factor test was adopted to check this bias. The results showed that nine factors had eigenvalues >1, while the variation explained by the first factor was 27.38%, which was less than the critical standard of 40%, indicating that there was no serious common method variance (23).

### Analysis of Differences in Life Meaning and Subjective Well-Being Among Older People in Different Groups

#### Analysis of gender differences in life meaning and subjective well-being among older people

**Figure 1:**
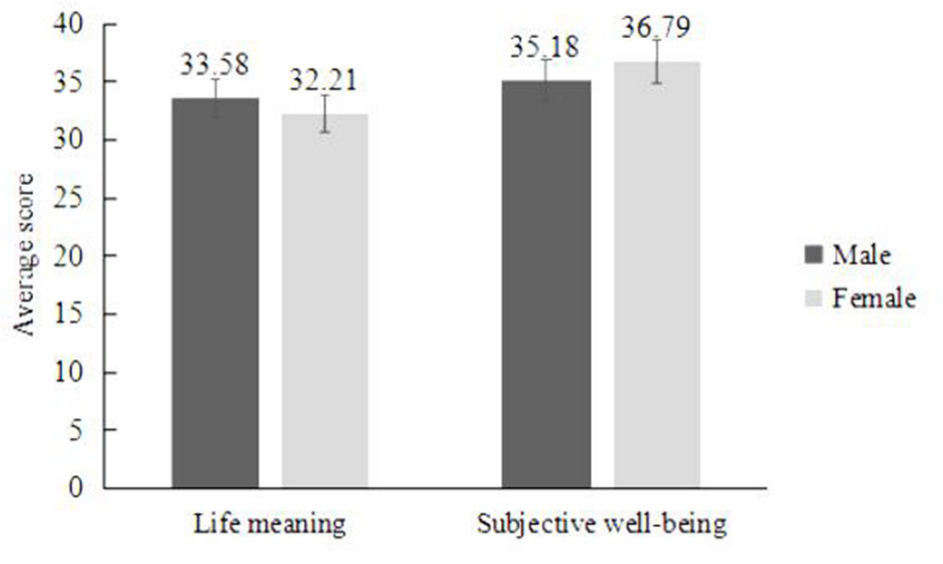
Comparison of differences between the life meaning and subjective well-being of older person of different genders. Gender differences existed in the respondents' perception of life meaning but not in the subjective well-being: men had a better perception of life meaning. The data on the bar chart are averages, and the vertical line represents the standard error.

An independent-samples *t*-test found significant gender differences among older people in terms of their perception of the meaning of life [*t*_(1,350)_ = 2.19, *SE* = 0.63, *p* < 0.05], suggesting that men had better perceptions of life meaning than women. There was no significant difference in the experience of subjective well-being among older people of different genders [*t*_(1,350)_ = 1.70, *SE* = 0.95, *p* > 0.05] (Figure 1).

#### Analysis of the Age Difference in Life Meaning and Subjective Well-Being Among Older People

**Figure 2:**
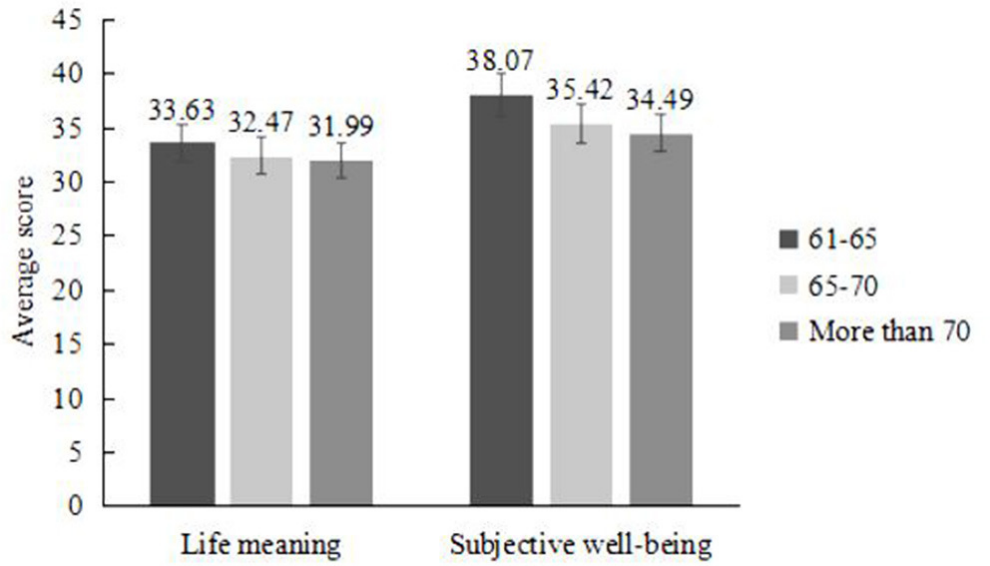
Comparison of scores of different scales among older people of different ages. Age differences existed in the subjective well-being of older people, but not in the life meaning. The subjective well-being of older individuals steadily declines with rising age. The data on the bar chart are averages, and the vertical line represents the standard error.

Using ANOVA, we found that older people of different ages exhibited no significant difference in life meaning perception [*F*_(2, 349)_ = 2.63, *partial* η^2^ = 0.02, *p* > 0.05]. However, older people of different ages significantly differed in their experience of subjective well-being [*F*_(2,349)_ = 5.69, *partial* η^2^ = 0.03, *p* < 0.01]. The *post hoc* analysis revealed a significant difference among the 61- to 65-year-old participants, 66- to 70-year-old participants, and over-70-year-old participants (*p* < 0.01), but there was no significant difference among the age groups (Figure 2). Specifically, as age increased, the experience of subjective well-being gradually decreased.

### Correlation Analysis of Each Variable

Table 1 shows significant correlations among life meaning, exercise identification, and subjective well-being (*r* = 0.48, *r* = 0.33, *p* < 0.01); among life meaning, amount of exercise and subjective well-being (*r* = 0.33, *r* = 0.33, *p* < 0.01); and between life meaning and subjective well-being (*r* = 0.33, *p* < 0.01). Life meaning, exercise identification, and subjective well-being are significantly correlated, and life meaning, amount of exercise, and subjective well-being are significantly correlated. Therefore, the relationship among the above variables can be further explored.

**Table 1:** Correlation analysis of the variables.

	**M ± SD**	**Life meaning**	**Exercise identification**	**Amount of exercise**	**Subjective well-being**
Life meaning	32.76, 5.81	1.00			
Exercise identification	8.35, 1.36	0.48^**^	1.00		
Amount of exercise	10.90, 8.21	0.33^**^	0.50^**^	1.00	
Subjective well-being	36.14, 8.76	0.33^**^	0.27^**^	0.49^**^	1.00

* *p < 0.05*,

** *p < 0.01*,

*** *p < 0.001, the same below*.

### Hierarchical Regression Analysis of Life Meaning and Subjective Well-Being

With subjective well-being as the dependent variable, gender and age were entered into the first layer, and life meaning was entered into the second layer. A hierarchical regression analysis was adopted to analyze the individual contribution of life meaning to subjective well-being. The results showed that life meaning had a significant positive impact on subjective well-being [β = 0.33, *t*_(3,348)_ = 6.38, *p* < 0.001]; thus, life meaning can positively predict subjective well-being, corresponding to our hypothesis. Excluding the influence of gender and age, the difference in subjective well-being explained by life meaning alone was 10%, which was statistically significant (Table 2).

**Table 2:** Hierarchical regression analysis of life meaning and subjective well-being.

**Level**	**DV**	**IV**	***R***	**Adjust *R*^**2**^**	***t***	**β**
First	Subjective well-being	Gender	0.17	0.03	0.43	0.03
		Age			−2.81^**^	−0.16
Second	Subjective well-being	Life meaning	0.36	0.12	6.38^***^	0.33

** *p < 0.01*,

*** *p < 0.001*.

### Mediating Effect Test of Exercise Identification

Exercise identification (W) was assumed to be the mediating variable between life meaning (X) and subjective well-being (Y). The mediating effect of exercise identification on life meaning and subjective well-being was tested according to the testing procedure for mediating variables (24).

The results (Table 3) showed that exercise identification and life meaning were still significant predictors of subjective well-being after the mediating variable of exercise identification was added, implying a partial mediating effect. The proportion of the mediating effect was 22% of the total effect.

**Table 3:** Examination of the mediating effect of exercise identification (W).

	**Standardized regression equation**	**Regression coefficient test**
First step	*y* = 0.33*x*	*SE* = 0.08, *t* = 6.48^***^
Second step	*w* = 0.48*x*	*SE* = 0.11, *t* = 10.27^***^
Third step	*y* = 0.26*x*	*SE* = 0.09, *t* = 4.48^***^
	+0.15*w*	*SE* = 0.33, *t* = 2.59^*^

* *p < 0.05*,

*** *p < 0.001*.

### Mediating Effect Test of Amount of Exercise

The amount of exercise (W) was assumed to be the mediating variable between life meaning (X) and subjective well-being (Y). The mediating effect of the amount of exercise on life meaning and subjective well-being was tested following the procedure for testing mediating variables (24).

The results (Table 4) showed that amount of exercise and life meaning were still significant predictors of subjective well-being after the mediating variable of amount of exercise was added, implying a partial mediating effect. The proportion of the mediating effect was 43% of the total effect.

**Table 4:** Examination of the mediating effect of amount of exercise (W).

	**Standardized regression equation**	**Regression coefficient test**
First step	*y* = 0.33*x*	*SE* = 0.08, *t* = 6.48^***^
Second step	*w* = 0.33*x*	*SE* = 0.07, *t* = 6.44^***^
Third step	*y* = 0.19*x*	*SE* = 0.07, *t* = 3.87^***^
	+0.43*w*	*SE* = 0.05, *t* = 8.97^***^

*** *p < 0.001*.

### Serial Multiple Mediation Pathway Analysis of Exercise Identification and Amount of Exercise

**Figure 3:**
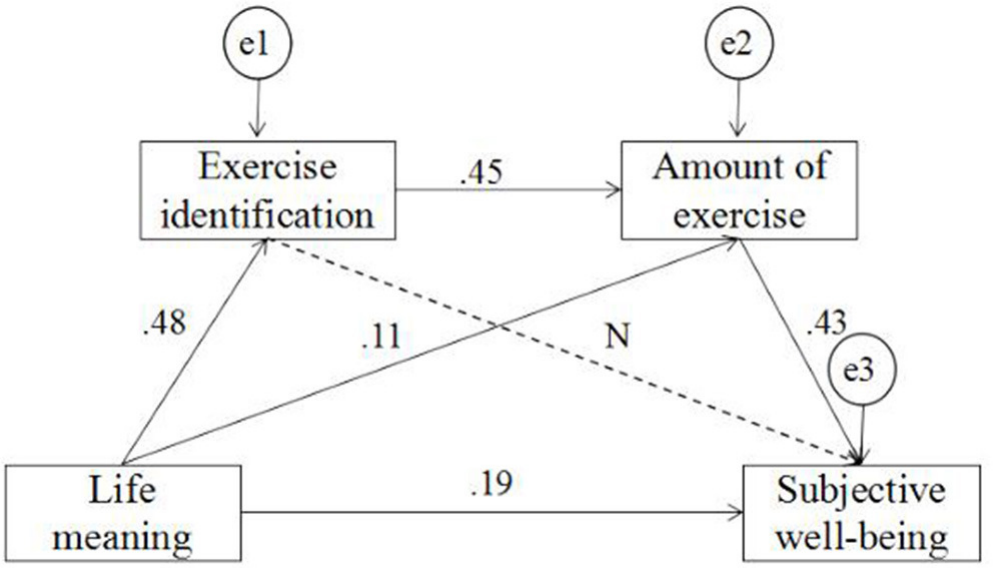
Serial multiple mediation pathways analysis. Solid arrows indicate the significant paths, and dashed arrows indicate not significant path (*N* = not significant). “e1, e2, e3” represent the error variables. Unstandardized coefficients are reported on the pathways.

Based on the above analysis, a pathway analysis chart of the saturation model of life meaning, exercise identification, amount of exercise, and subjective well-being was established. Since the regression coefficient between exercise identification and subjective well-being was not significant (*p* = 0.33>0.05), the path from exercise identification to subjective well-being was removed, and the path analysis chart was reestablished (Figure 3). The fitting results showed that the model had a good chi-square effect *i*χ^2^*/df* = 0.94, *GFI* = 0.99, *AGFI* = 0.98, *NFI* = 0.99, *IFI* = 1.00, *CFI* = 1.00, *RMSEA* = 0.00), and the overall fit was satisfactory.

The bootstrap method was used to test the serial multiple mediation effect of “exercise identification—amount of exercise” to determine the influence of life meaning on subjective well-being over 5,000 iterations. The serial multiple mediation effect was 0.09, and the 95% confidence interval was [0.14, 0.29], excluding 0, indicating that the serial multiple mediation effect was significant and the effect amount was 27%. These results verified our hypothesis. In addition, the direct effect of meaning of life on subjective well-being was 58%, and the effect of the amount of exercise was 15% (Table 5).

**Table 5:** Mediation effect and effect size of the serial multiple mediation model.

**Effect**	**Mediation pathways**	**Effect of value**	**Effect size**
Direct effect	Life meaning → Subjective well-being	0.19	58%
Indirect effect	Life meaning → Exercise identification → Amount of exercise → Subjective well-being	0.09	27%
	Life meaning → Amount of exercise → Subjective well-being	0.05	15%
Total effect		0.33	100%

To test whether the serial multiple mediation pathway diagrams were universally applicable, we conducted a multigroup analysis of the above model according to gender (Table 6). The results showed that the unconstrained model was equal to the model of the structural weight; thus, there was no significant difference in the factor load values of the older person's model for different genders. Comparison of the structural covariance model and the structural weight model found no significant difference; thus, gender played a moderate role in the model. Regarding the above model explaining older women, each pathway coefficient in the model was significant (*p* < 0.001). Among older men, the path coefficients from life meaning to amount of exercise and from life meaning to subjective well-being were not significant (*p* > 0.05). A moderate effect appeared in the two pathways from life meaning to amount of exercise and from life meaning to subjective well-being, and this model was more effective in explaining older women.

**Table 6:** Fit index of the gender group analysis model comparison.

**Indicators**	***χ2/df***	***IFI***	***TLI***	***RFI***	***NFI***	***AGFI***	***RMSEA***
Unconstrained model	4.42	0.98	0.89	0.86	0.98	0.88	0.09
Structural weights model	2.11	0.98	0.96	0.93	0.96	0.94	0.06
Structural covariances model	2.86	0.96	0.94	0.91	0.94	0.92	0.07

## Discussion

### Gender and Age Differences in Life Meaning and Subjective Well-Being Among Older People

There were significant differences in the perception of life meaning among older people of different genders, with men having a better perception of life meaning than women. However, this conclusion is controversial. A previous study has found that women had higher life meaning scores than men (25). This difference may be related to differences in the lifestyles and cultures of people in different regions. As in this study only older persons in Yueyang, Hunan Province, were investigated, further studies should be conducted in different regions of China.

There are significant differences in the experience of subjective well-being among older people of different ages, which are manifested in the gradual decrease in the experience of subjective well-being with increasing age. With increasing age, the living ability and living conditions of older people gradually decline, and their subjective well-being decreases accordingly. He (26) studied the subjective well-being of older people of different ages of the Naxi nationality (an ethnic minority in China), and the results were consistent with those reported in this paper.

### Positive Predictive Effect of Life Meaning on the Subjective Well-Being of Older People

A significant positive correlation exists between life meaning and subjective well-being, and life meaning can positively predict the subjective well-being of older people, which is consistent with the research conclusions reported by Jin et al. (27) concerning the relationship between life meaning and subjective well-being. Chen (28) noted that the influence of life meaning on subjective well-being can be divided into direct and indirect ways. (1) Older people with a higher perception of life meaning still search for worthy goals and strive to achieve these goals in their later years (29). Older people with a higher perception of life significance still search for valuable goals and strive to achieve them. The process of striving to achieve goals is valuable and pleasing. When they reach these goals, older people experience satisfaction and subjective happiness. (2) The meaning of life indirectly affects subjective well-being through other mediating factors. Researchers (30) investigated older people in 250 different communities, and the results showed that optimism plays an intermediary role in the influence of life meaning on subjective well-being. In this study, exercise identification and amount of exercise also played an intermediary role in the influence of life meaning on subjective well-being. However, as this study only adopted a cross-sectional design, causality cannot be established. Thus, it is expected that further study should evaluate the relationship longitudinally.

### Serial Multiple Mediation Effect of Exercise Identification and Amount of Exercise

A significant correlation exists among life meaning, exercise identification, and subjective well-being. Exercise identification has a partial mediating effect on the influence of life meaning on subjective well-being, with a mediating effect of 22%. A significant correlation exists among life meaning, amount of exercise, and subjective well-being. The amount of exercise has a partial mediating effect on the influence of life meaning on subjective well-being, with a mediating effect of 43%. When the serial multiple mediation model was further established, it revealed that exercise identification and the amount of exercise play a serial multiple mediation role in the relationship between life meaning and subjective well-being.

Relevant studies have found correlations between life meaning and the amount of exercise (31, 32). In addition, Liu (33) noted that there are various ways to express life meaning, and exercise is one of these ways. Improving the understanding of life meaning can also promote exercise. Chen et al. (34) found that physical exercise can significantly improve the subjective well-being of older people. Wang et al. (35) noted that the overall mental health status and subjective well-being of older people who participate often in physical exercise were significantly better than those of older people in the non-exercise group, likely because physical exercise helps older people maintain a healthy state of body and mind by prompting them to move. Additionally, exercise is generally performed in public, thus increasing older people's opportunity to interact with others. Exercise is also a good opportunity to enhance emotional communication. It is beneficial for older people to cultivate friendships with like-minded people because as loneliness decreases, subjective well-being increases. Furthermore, some studies have noted that paying attention to physical exercise can render older people more energetic and more efficient (36), which may explain why older people who have a good understanding of life meaning participate in more physical exercise. Therefore, life meaning can be changed not only at the cognitive level to improve the subjective well-being of older people but also at the practical level; the pursuit of life meaning can lead to an increase in the amount of exercise and improve the subjective well-being of older people.

Xu and Wang (37) noted that exercise identification plays an important role in predicting exercise behavior. Simultaneously, physical exercise can promote the subjective well-being of older people and improve their quality of life (38). Therefore, the perception and pursuit of life meaning lead to valuing physical exercise, recognizing the significance of physical exercise, improving the amount of exercise, and ultimately increasing the subjective well-being of older people.

In the serial multiple mediation model, gender played a moderating role in the influence of life meaning on the amount of exercise and subjective well-being. Specifically, among older women, life meaning has a significant effect on the amount of exercise and subjective well-being. Future studies should examine whether it is possible to enhance life meaning through interventions that influence exercise identification and the amount of exercise.

## Data Availability Statement

All datasets generated for this study are included in the article/Supplementary Material.

## Ethics Statement

The studies involving human participants were reviewed and approved by the Kinetic Science Experiment Ethics Committee of Beijing Sport University. Participation was totally voluntary, and responses were anonymous. All the information collected was stored securely. A participant information sheet was provided at the start of the data collection. Completion of the questionnaire indicated consent. The patients/participants provided their written informed consent to participate in this study. Written informed consent was obtained from the individual(s) for the publication of any potentially identifiable images or data included in this article.

## Author Contributions

YY conducted data collection. QZ performed data analysis, carried out the bulk of the literature review, and manuscript writing. G-LZ participated in checking methods, results, guiding QZ during the data analysis, and played an editorial role when it came to writing up the research study. All authors contributed to the article and approved the submitted version.

## Conflict of Interest

The authors declare that the research was conducted in the absence of any commercial or financial relationships that could be construed as a potential conflict of interest.
