# Supplementary material for: Influence of Life Meaning on Subjective Well-Being of Older People: Serial Multiple Mediation of Exercise Identification and Amount of Exercise
Source: Front Public Health. 2021 Jul 8;9:515484. doi: 10.3389/fpubh.2021.515484 (PMC8295607; doi:10.3389/fpubh.2021.515484)
Supplement: Supplementary file 2 [file Table_2.DOCX]

**1.Exercise identification questionnaire**

This is a questionnaire to find out how you feel about physical activity, with a four-point score.

a1. Your attitude towards physical exercise:

(1)Like it very much (2)Like (3)General (4) Dislike

a2. Would you like to take part in physical exercise?

(1)Very willing to (2)Willing to (3)Dose not matter (4)Don't want to

a3. Do you think it’s good for your health to take part in sports regularly ?

(1)Very useful (2)Useful (3)Not clear (4)Useless

**2.Physical activity habits questionnaire**

b1. Do you take part in sports exercises?

(1)To participate in. (2)Do not participate in.

b2. The reason why you participate in physical exercise is (multiple choices):

(1)Good for your health. (2)Making friends. (3)Kill time. (4)Hobbies.

(5)The atmosphere in the activity area is good. (6)To make life pleasant. (7)Others (please specify).

b3. The reason why you do not participate in physical exercise is (multiple choices):

(1)Activities do not suit one's interests. (2)The activity place condition is not good. (3)Have no time. (4)The atmosphere in the venue is not good. (5)No activity space. (6)Not interested in sports. (7)The activity is not organized and managed. (8)Others (please specify).

b4. How many times do you take part in physical exercise every week?

(1) More than 3 times a week. (2) Once or twice a week. (3)Once or three times a month. (4) Once in a while. (5) Never exercise.

b5. How long do you exercise every time?

(1) Once every 30 minutes. (2)30 to 60 minutes. (3) Over 60 minutes. (4) Not sure.

b6. When do you usually take part in physical exercise?

(1)In the morning. (2)At noon. (3)In the afternoon. (4) Evening.

b7. Some of the sports you often take part in are (multiple choices):

(1)Broadcast gymnastics. (2)Running. (3)Take a walk. (4)Tennis, table tennis, badminton, billiards and other small balls. (5)Basketball, volleyball, football. (6)Qigong. (7)Tai chi chuan. (8)Swimming. (9)Fitness dance. (10)Poker, chess. (11)Fishing. (12)Others (please specify).

b8. The place where you participate in physical exercise is usually (multiple choices):

(1)Community public sports venues. (2)Open Spaces and squares in the community. (3)Community green space. (4)Park. (5)Charging for sports venues. (6)Indoors. (7)Courtyard balcony. (8)Others (please specify).

b9. Do you think the facilities in your community can meet your needs for sports activities?

(1)Absolutely. (2)To be able to. (3)In general. (4)Can't. (5) Not at all. (6)It doesn't matter.

b10. Do you want someone to coach you while you are participating in sports activities?

(1)Really hope. (2)Hope. (3)It doesn't matter. (4)Don't want to.

b11. Is there a mentor at your location?

(1)Yes, so his identity is (a)Social sports instructor. (b)Street sports association cadres. (c)In-service professional sports staff. (d)The emeritus and retired. (e)Amateur sports activist. (f)Other personnel.

(2) No.

b12. Which of the following forms of sports do you take part in (multiple choices):

(1)Participate in sports activities or competitions organized by community sports organizations. (2)For-profit sports and fitness clubs. (3)Join a community sports club. (4)Individual freedom of movement. (5)Morning and evening exercise activity station. (6)Don't participate. (7)The other.

b13. The amount of exercise you take out each time is generally controlled at:

(1)The body does not sweat. (2)Sweat slightly. (3)Break a sweat. (4)Don't control. (5)Exhausted.

b14. Do you have an exercise prescription for yourself?

(1)Yes. (2)No.

b15. If not, would you like to have an exercise prescription that suits you?

(1)Really hope. (2)Hope. (3)It doesn't matter. (4)Don't want to.

b16. Are you satisfied with your present life?

(1)Satisfied with it. (2)In general. (3)Not satisfied.

b17. What do you think of your health?

(1)Very good. (2) Good. (3)In general. (4) Poor.

b18. Do you have any disease?

(1)Yes. (2)No.

b19. Which disease do you have?

b20. How long has the disease been?

b21. Are you afraid of getting sick?

(1)Yes. (2)No.

b22. After the exercise, you feel the biggest benefit?

(1)Energetic. (2)Feel better. (3)Reduced incidence. (4)Live life to the fullest.

(5)More friends. (6)Get noticed. (7)I like sports more. (8)The other.

b23. At present, the urgent problems to be solved in the process of organizing and managing physical exercise for the elderly are as follows:

(1)Publicize the knowledge of physical exercise. (2)Open site facilities. (3)Organize special person to instruct. (4)Teach motor skills. (5)Establish a sports club or sports association. (6)Create an atmosphere for physical exercise. (7)The other.

b24. What is your favorite sport to take part in? (single choice)

(1)Basketball. (2)Volleyball. (3)Football. (4)Table tennis. (5)Badminton.

(6)Running (7)Wushu. (8)Yoga. (9)Tae kwon do. (10)The other._____

b25. How hard do you do physical exercise? (If the frequency of several activities is similar, please choose the activity with the maximum exercise intensity.)

(1)Light exercise (such as walking, doing radio exercises).

(2)Low-intensity, less intense exercise (such as playing table tennis, jogging, etc for recreational purposes).

(3)Moderately intense and sustained exercise (e.g. cycling, running, playing table tennis).

(4)Intense, but not prolonged, exercise that causes shortness of breath and a lot of sweating (playing badminton, basketball, etc.).

(5)Intense, prolonged exercise that causes shortness of breath and a lot of sweating (e.g. running, swimming, etc).

b26. How many minutes do you spend in each of these activities?

(1)Under 9 minutes. (2)10 to 19 minutes. (3)20 to 29 minutes.

(4)30 to 59 minutes. (5)More than an hour.

b27. How many times a month do you do these sports?

(1)Less than once a month. (2)Two to three times a month. (3)Once or twice a week.

(4)Three to five times a week. (5)About once a day.

b28. How long has it lasted since you participated in regular physical activity?

(1)Less than 1 year. (2)1 to 2 years. (3) 3-4 years. (4)More than 5 years.

b29. What kind of organization do you take part in physical exercise?

(1)Alone. (2)With a partner. (3) Family. (4)School organization.

b30. If no one is urging you, will you voluntarily participate in physical exercise?

(1)Yes. (2)No.

**3. University of Newfoundland Happiness Scale**

We'd like to know something about your life. If it suits you, answer “Yes”. If it doesn't, answer “No”. If you don't know, answer “Don't know”. In recent months, you have felt:

c1. Satisfied to the hilt?

c2. In a good mood?

c3. Particularly satisfied with your life?

c4. Very lucky?

c5. Trouble?

c6. Annoyed or alienated?

c7. Worried or very unhappy?

c8. Worried because of don't know what will happen in the future.

c9. Feeling that your life situation has become difficult.

c10. Generally speaking, life situations become satisfying.

c11. This is the worst period of my life.

c12. I am as happy as when I was young.

c13. Most of what I do is boring or monotonous.

c14. What I do interests me as before.

c15. When I look back on my life, I feel quite satisfied.

c16. Everything gets worse with age.

c17. Do you feel lonely?

c18. Something is bothering me this year.

c19. If you could live where you want to live, would you like to live there?

c20. Sometimes I feel bored living.

c21. I am as happy as when I was young.

c22. Most of the time I find life hard.

c23. Are you satisfied with your present life?

c24. My health is the same as or better than that of my peers.

**4.Chinese life meaning scale**

First, please take a moment to think about what makes you feel important in your life. Then, make a choice based on how well the following description fits your situation, and circle "0" or check "√" on the selected number. Please answer as accurately and truthfully as possible. The following questions are highly subjective, and each person's answer will be different. There is no right or wrong answer.

“1” = Strongly disagree

“2” = Basically disagree

“3” = Somewhat disagree

“4” = Not sure

“5” = Somewhat agree

“6” = Basically agree

“7” = Strongly agree

d1.I know the meaning of my life

1 2 3 4 5 6 7

d2.I'm looking for something to fill my life with meaning

1 2 3 4 5 6 7

d3.I'm always looking for a purpose in life

1 2 3 4 5 6 7

d4.I have a definite purpose in life

1 2 3 4 5 6 7

d5.I know exactly what makes my life meaningful

1 2 3 4 5 6 7

d6.I have found a satisfying purpose in life

1 2 3 4 5 6 7

d7.I've been looking for something that makes my life feel important

1 2 3 4 5 6 7

d8.I'm looking for a purpose and a "mission" in my life

1 2 3 4 5 6 7

d9.I don't have a clear goal in life

1 2 3 4 5 6 7

d10.I am searching for the meaning of my life

1 2 3 4 5 6 7
